# Protein synthesis, degradation, and energy metabolism in T cell immunity

**DOI:** 10.1038/s41423-021-00792-8

**Published:** 2022-01-04

**Authors:** Julia M. Marchingo, Doreen A. Cantrell

**Affiliations:** Cell Signalling and Immunology Division, School of Life Sciences, University of Dundee, Dundee, DD1 5EH UK

**Keywords:** T lymphocyte, Protein Translation, Proteomics, Immunometabolism, Protein degradation, T cells, TOR signalling, Proteolysis, Lymphocyte activation, Cell growth

## Abstract

T cell activation, proliferation, and differentiation into effector and memory states involve massive remodeling of T cell size and molecular content and create a massive increase in demand for energy and amino acids. Protein synthesis is an energy- and resource-demanding process; as such, changes in T cell energy production are intrinsically linked to proteome remodeling. In this review, we discuss how protein synthesis and degradation change over the course of a T cell immune response and the crosstalk between these processes and T cell energy metabolism. We highlight how the use of high-resolution mass spectrometry to analyze T cell proteomes can improve our understanding of how these processes are regulated.

## Introduction

Upon pathogen recognition, naive T cells rapidly increase energy production and produce a large number of new messenger RNA (mRNA) transcripts and proteins. Activated T cells also undergo massive growth, doubling to quadrupling in size over a 1–2 day period [1–3], followed by a rapid series of cellular divisions every 6–12 h [4]. This complete overhaul of the cellular transcriptome and proteome results in substantial remodeling of multiple pathways involved in cellular metabolism and protein synthesis, including key energy production pathways governing mitochondrial, glycolytic and lipid metabolism; pathways important for the synthesis of biomolecules, such as nucleotides, amino acids and fatty acids; and ribosomal protein production machinery, as outlined in several proteome resource studies [2, 5–7]. These changes support the massive clonal expansion of pathogen-specific T cells and their differentiation into effector cell subsets, where they function as protein production factories for effector molecules, including inflammatory cytokines and cytolytic granzymes. After pathogen clearance, the effector T cell population contracts, whereas memory T cells persist in a quiescent state, ready for reactivation if reinfection occurs.

The protein production and degradation that drive T cell activation are energy- and resource-intensive processes that are shaped by the T cell environment and the availability of key nutrients [8–10]. In this review, we will primarily focus on how immune-activated T cells control protein synthesis. We will also discuss how protein synthesis and degradation are regulated by T cell energy metabolism. Throughout the review, we will highlight how information in publicly available T cell proteomics datasets (listed in Table 1) can enhance our understanding of protein synthesis control and T cell metabolic pathways.

**Table 1:** List of highlighted proteomics resources

Reference	Mass spec technique	Cell type/stimulation condition	Information available
Damasio [1]	TMT-labeled fractionated DDA	Mouse T cells, 3 biological replicates: —CD8 Naive —CD8 24 h peptide treatment —CD8 24 h peptide + Mek inhibitor treatment	Whole cell proteome
			Copies/cell
			Fold change, statistics
Howden* [2]	TMT-labeled fractionated DDA	Mouse T cells, 3 biological replicates: —CD8 Naive —CD4 Naive —CD8 treated for 24 h with peptide + IL2/IL12 —CD4 treated for 24 h with peptide/APC + IL2/IL12 —CTLs in vitro generated with IL2/12 —Th1 in vitro generated with IL2/12 —All activated T cell subsets treated for 24 h +/− rapamycin	Whole cell proteome
			Copies/cell
			Concentration (nM)
			Mass (pg)/cell
			Fold change, statistics
			website: immpres.co.uk
Hukelmann [33]	SILAC-labeled fractionated DDA	Mouse T cells, 3 biological replicates: —CTLs in vitro generated with IL2/IL12+/− rapamycin for the final 48 h	Whole cell proteome
			Copies/cell, fold change, statistics
Ma [36]	TMT-labeled fractionated DDA	Mouse T cells, 5 biological replicates: —Naive and activated OT1 CD8 T cells from day 2.5 of a *Listeria*-OVA infection	Whole cell proteome
			Summed peptide intensities
Marchingo [3]	Label-free fractionated DDA	Mouse T cells, 3 biological replicates: —CD8 Naive —CD8 WT treated for 24 h with αCD3/αCD28 —CD8 MycKO treated for 24 h with αCD3/αCD28 —CD4 Naive —CD4 WT treated for 24 h with αCD3/αCD28 —CD4 MycKO treated for 24 h with αCD3/αCD28 —CD4 WT treated for 24 h with αCD3/αCD28 + IL2/IL12 —CD4 Slc7a5KO treated for 24 h with αCD3/αCD28 + IL2/IL12	Whole cell proteome
			Copies/cell
			Mass (pg)/cell
			Fold change, statistics
			website: immpres.co.uk
Rieckmann [42]	Label-free single-shot DDA	Human T cells and other hematopoietic lineages, 4 biological replicates: —CD4/CD8 Naive, memory T cells, and effector memory T cells at steady state or activated for 48 h with αCD3/αCD28 and then cultured for 48 h with IL2 —CD4 Th1, Th2, and Th17 at steady state	Whole cell proteome
			Summed peptide intensities, LFQ, iBAQ, copies/cell
			website: immprot.org
Rollings [37]	Label-free fractionated DDA	Mouse T cells, 3 biological replicates: —In vitro IL2-expanded CTLs treated for 24 h +/− IL2 —In vitro IL2-expanded CTLs treated for 24 h +/− Jak1/3 inhibitor	Whole cell proteome
			Copies/cell
			Fold change, statistics
Ross [38]	SILAC-labeled Fractionated DDA	Mouse T cells, 3 biological replicates: —In vitro IL2-expanded CTLs treated overnight in IL12 only then +/− IL2 for 15 min —In vitro IL2-expanded CTLs treated for 30 min or 4 h with Jak1/3 inhibitor —In vitro IL2-expanded CTLs treated for 4 h with Src family inhibitor	Phosphoproteome
			SILAC ratio of summed peptides, stats
Tan* [6]	TMT-labeled fractionated DDA	Mouse T cells, 2 biological replicates —Naive CD4 T cells —αCD3/αCD28-activated WT CD4 T cells after 2, 8, or 16 h —αCD3/αCD28-activated Raptor KO CD4 T cells after 2 or 16 h	Whole cell proteome and phosphoproteome
			Summed peptide intensities, fold change, statistics
Wolf* [7]	SILAC-labeled Single-shot DDA	Human T cells, 3–7 biological replicates: —Naive/Memory CD4 T cell protein turnover (SILAC labeling time course) —Naive CD4 T cells treated for 24 h (control, + translation inhibitor, or + translation and proteasome inhibitor) —Naive or αCD3/αCD28-activated CD4 T cells at 6, 12, 24, 48, 72, 96, 120, and 144 h —RNAseq data for Naive and αCD3/αCD28-activated CD4 T cells at 6 and 24 h	Whole cell proteome
			Turnover kinetics (naive, memory), copies (CHX experiment)
			LFQ, copies (activation time course)
			Estimated transcript/cell
			website: immunomics.ch

Resource papers are studies of primary human or mouse T cells. For whole cell proteome studies, authors included intensity/copy number information as well as fold changes and statistics as Supplementary Tables or on Websites, therefore making data accessible for flexible interrogation by all readers without the need to reanalyze raw files.

An asterisk indicates studies that are particularly useful for understanding changes in protein expression over time.

## General overview of protein synthesis

**Fig. 1:**
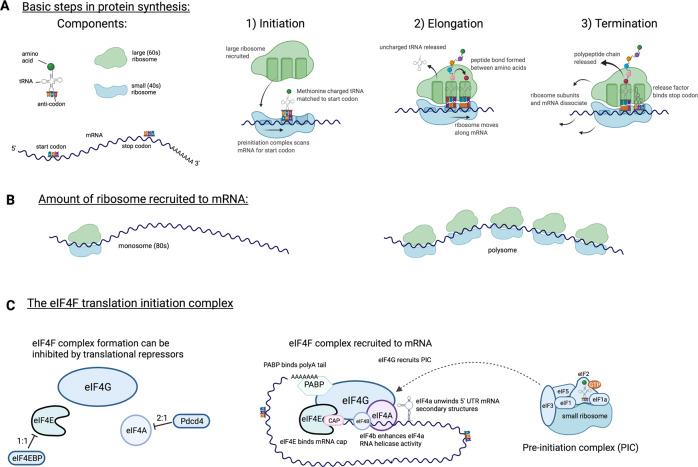
Features of protein synthesis. **A** Basic schematic of the three major steps in protein synthesis: initiation, elongation, and termination. **B** A single ribosome recruited to mRNA is referred to as a monosome; when multiple ribosomes are simultaneously recruited to mRNA, the structure is called a polysome. **C** (Left) The translational repressors eIF4EBP1-3 and PDCD4 can prevent eIF4E and eIF4A, respectively, from binding to the eIF4G scaffold protein. (middle) Schematic of eIF4F recruitment to mRNA. (right) Schematic of the preinitiation complex

Protein synthesis involves linking amino acids into a polypeptide chain in the order specified by the nucleotide sequence of a mRNA transcript. A simplified diagram of protein synthesis is provided in Fig. 1a. The ribosome, a macromolecular complex composed of ribosomal RNA (rRNA) and many ribosomal proteins, is the molecular machine that facilitates protein synthesis. Amino acids are brought to the ribosome by transfer RNAs (tRNAs). For each codon in mRNA that is translated into protein, there is at least one tRNA that contains the matching anti-codon and thus carries the corresponding amino acid. Each ribosome consists of two subunits that have distinct functions and must be brought together for protein synthesis to occur. The small (40 S) subunit binds the mRNA and contains the site where the tRNA anti-codon is matched to the complementary mRNA sequence. The large (60 S) subunit contains the site where the peptide bonds between amino acids brought to the ribosome by tRNA are formed, ultimately generating the polypeptide chain that is subsequently folded to form the final protein structure. As illustrated in Fig. 1b, mRNA can be associated with a single complete (80 S) ribosome, referred to as a monosome, or translated simultaneously by multiple ribosomes in a structure called a polysome. Once the ribosome reaches a termination codon, for which there is no matching tRNA, translation terminates, and the ribosomal subunits dissociate and are recycled for use in another round of translation [11, 12].

Translation of an mRNA sequence into a protein is frequently described as involving three main steps: translation initiation, elongation and termination (Fig. 1a) [11–13]. A major point of protein synthesis regulation is at translation initiation. All mammalian mRNAs are marked with a methylated guanosine cap at their 5' end during RNA polymerase II-mediated transcription. The cap structure is critical for binding proteins that control translation initiation by recruiting mRNA to the ribosome or translation repression by preventing ribosome interaction [13–15]. Most mRNAs in mammalian cells are recruited for translation via the eIF4F complex, a heterotrimer consisting of the proteins eIF4E, eIF4G and eIF4A [12, 13]. As illustrated in Fig. 1c, eIF4E binds the mRNA cap to recruit the mRNA for translation. Many mRNAs have secondary structures in their 5' untranslated regions (UTRs). When eIF4A is bound to eIF4G, it mediates the unwinding of these secondary structures through its RNA helicase activity, which can be enhanced by eIF4B and eIF4H. eIF4G acts as the scaffold protein, enhancing eIF4A activity and bringing eIF4E-bound mRNA to the preinitiation complex (PIC). The PIC contains the small ribosomal subunit and a series of other translation initiation factors, including GTP-bound eIF2, which mediates the binding of initiator tRNA. eIF4G also recruits polyadenylate binding protein, which interacts with the 3' mRNA end, allowing transcript circularization and enhancing protein translation. Once recruited, the PIC scans the mRNA for the translational start site, indicated by an AUG codon encoding the amino acid methionine. Once the start codon is identified and paired with methionine-charged tRNA, eIF2-GTP is hydrolyzed and dissociates from the PIC, and the large ribosome is recruited. At this point, protein elongation commences [12, 13].

## How protein synthesis capacity and activity change during a T cell immune response

**Fig. 2:**
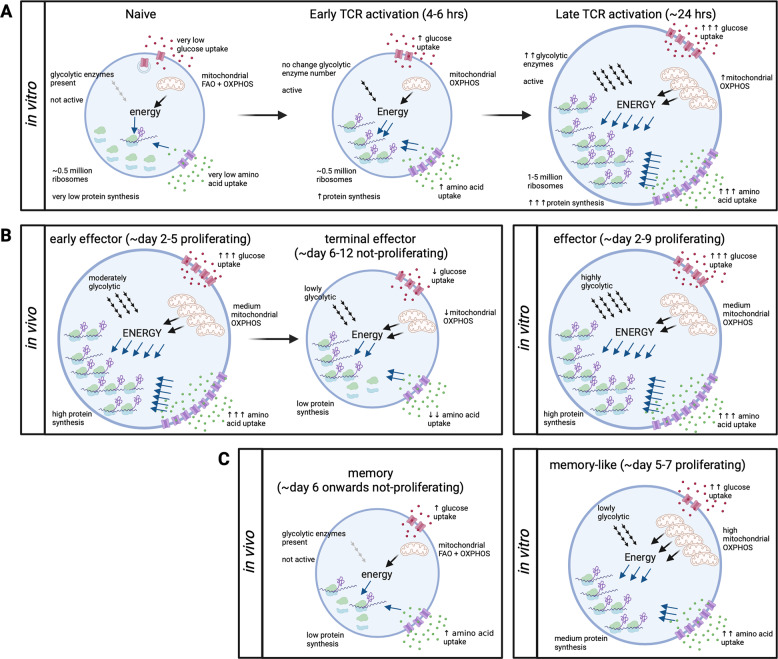
Protein synthesis and energy production during in vitro and in vivo T cell responses. Changes in amino acid uptake (via purple transporters); glucose uptake (via red transporters); ribosome assembly and protein synthesis; and energy (ATP) production via glycolysis (diagonal 4× arrows) and mitochondrial oxidative phosphorylation (OXPHOS) or fatty acid oxidation (FAO) in **A** ex vivo naive and in vitro 6 and 24 h TCR-activated T cells; in vivo and in vitro generated **B** effector T cells; and **C** memory T cells. **A** Naive T cells have very low nutrient uptake, protein synthesis, and energy production. T cell activation increases protein synthesis and energy production by increasing nutrient uptake and engaging preformed protein machinery before further increasing the expression of nutrient transporters, metabolic machinery, and ribosomes to support large-scale cell growth. **B**, **C** In vivo activated cells maintain a high growth phenotype only while they proliferate, whereas nutrient uptake, energy production, and protein synthesis are reduced when they terminally differentiate into effector or memory T cells. In vitro-generated T cells maintain a high growth phenotype for the entire culture period, with effector (IL2-cultured) T cells exhibiting a higher pro-growth phenotype than memory-like (IL15-cultured) T cells

The following sections will outline how protein synthesis changes as naive T cells respond to pathogens and differentiate into effector and memory cells. A schematic of the changes in nutrient uptake, protein synthesis, and cell metabolism is provided in Fig. 2.

### Naive T cells

In naive T cells, the rate of protein synthesis is low [16–18] but not zero, and naive T cells synthesize a surprisingly large number of proteins [19]. A recent study measuring protein turnover revealed that 19% of protein species detected in naive human CD4 T cells were at least partially degraded and regenerated over a 24-h period in culture [7]. In this study, estimates of individual protein turnover rates in naive T cells varied widely; some proteins had renewal half-lives of <1 h (such as ETS1), whereas others had renewal half-lives of >200 h (e.g., GAPDH) [7]. Critically, the continued synthesis of new proteins in naive T cells is functionally important. Many transcription factors and survival and homing proteins (e.g., IL7R, CD62L, TCF1, and FOXO1) required for naive T cell maintenance are rapidly turned over [7], consistent with the result that naive T cell quiescence is actively maintained [20, 21].

One estimate by Wolf et al. is that naive T cells translate ~60,000 proteins/min [7]. These findings of substantial translation in naive T cells are supported by a ribosome profiling study that found that ~23% of mRNAs were enriched for ribosome-protected fragments in naive mouse CD4 T cells [19]. Naive T cells have a sizeable pool of ribosomal subunits; proteomics and rRNA measurements have yielded estimates of ~400,00–700,000 ribosomes per cell [2, 7]. However, the majority of these ribosomes are not thought to be actively engaged in translation. The translationally active ribosome pool is in polysomes (Fig. 1b), but multiple polysome profiling studies in naive T cells have reported the presence of very few polysomes, with most RNA in subpolysome fractions [16, 17, 22, 23]. Whether protein synthesis in naive T cells occurs in monosomes or in the few polysomes present is not known. However, recent work in yeast and rat neurons has indicated that contrary to dogma, monosomes can substantially contribute to cellular protein synthesis [24, 25].

### T cell activation

It has been known since the 1960s that stimulated leukocytes increase in size prior to entering the cell cycle [26]. During a T cell response, this ‘blasting’ phenomenon is triggered by T cell receptor (TCR) signaling, which drives a major increase in protein synthesis and large-scale proteome remodeling before the first cell division. Initial changes in protein synthesis can occur rapidly, with thousands of copies of hundreds of new protein species being produced within a few hours of activation [5–7]. Wolf et al. have estimated that within 6 h of TCR activation, T cells are translating ~300,000 proteins/min, a 5-fold increase compared to the rate in naive T cells [7]. Generally, substantial cell growth and the amplification of protein synthesis do not begin until at least 8–12 h post TCR triggering in vitro [5–7, 27], and large increases in size have been observed by 24 h post activation both in vitro and in vivo [28]. Wolf et al. estimated that by 24 h after TCR activation in vitro, T cells are synthesizing ~800,000 proteins/min. Protein synthesis continues to increase for 1–2 days as cells prepare to commence a rapid division phase.

Measurable T cell growth corresponds with the increased expression of ribosomal proteins and other translational machinery [5–7, 23]. By a series of elegant calculations using time course proteomics data, Wolf et al. estimated that ribosomes translate ~4 amino acids/sec at both 6 and 24 h post TCR stimulation [7], indicating that the increased protein synthesis capacity of immune-activated T cells is likely due to higher quantities of ribosomes and translational machinery and not changes in ribosome activity [7, 29]. In vitro proteomics studies suggest that protein content increases by an estimated 3–4-fold after 24 h of TCR activation, and ~50–70% of the proteome is altered compared to that in naive T cells. The majority of these changes are increases, with 50–60% of total proteins detected being newly expressed or upregulated by more than 1.5-fold [1–3].

Some proteome remodeling during T cell activation requires de novo mRNA production, including the expression of mRNA encoding transcription factors, cytokines, and chemokines, which are critical for immune cell activation and function [7, 28, 30, 31]. Indeed, the immune activation of T cells initiates large-scale remodeling of the transcriptome, with numerous mRNAs being created or destroyed. A modest 1.4-fold increase in total mRNA content was observed 6 h post immune activation in vitro [7], with a more substantial ~5–10-fold increase within 20–24 h post activation [7, 32]. Despite this, mRNA quantity only moderately correlates with protein expression levels during T cell activation and in differentiated cytotoxic T lymphocytes (correlation coefficient ranging from 0.4 to 0.65) [6, 7, 33]. Preformed transcripts, which are present but not translated in naive T cells, contribute to this disconnect, although they encode only ~3% of detected protein species [7]. Such preformed transcripts include those for important proteins such as the AP-1 transcription factors JUNB and FOS and the glucose transporters SLC2A1 and SLC2A3 (GLUT1 and 3, respectively) [7, 17]. Other translational and posttranslational mechanisms, such as translational control by other RNA-binding proteins (RBPs), differences in mRNA and protein modification and stability, and temporal differences between mRNA and protein expression, also contribute substantially to the mRNA/protein discrepancies observed in T cells, as discussed elsewhere [34, 35].

### Effector and memory T cells

The magnitude of the increase in global protein synthesis during effector T cell differentiation is dependent on the system and time point studied, as outlined in Fig. 2b. During an in vivo CD8 T cell response, TCR-activated T cells increase in mass and remain large for a period of several days while they rapidly proliferate [4, 16, 28]. During this time, they show high mRNA and protein expression of translational machinery [28, 36], a high polysome content and high amounts of protein synthesis [16]. By the peak of T cell expansion (a time point regularly assayed to assess differentiated effector and memory phenotypes), the cells are no longer dividing, and the differentiated effector T cells are small, with a protein synthesis rate lower than at earlier activation timepoints but still higher than that in naive T cells [4, 16, 28]. These in vivo changes in protein synthesis rates in effector CD8 T cells temporally correlate with the translational repression of mRNA encoding ribosomal subunits and translation initiation factors. Hence, the capacity for protein synthesis is actively controlled during a T cell response and appears to be closely linked to both the initiation and cessation of T cell division [16].

When effector CD4 and CD8 T cells proliferate and differentiate in vitro in nutrient-rich media in the presence of inflammatory cytokines, they can sustain the high levels of expression of ribosomes and translational machinery. As a consequence, in vitro-generated effector T cells remain extremely translationally active with high levels of protein synthesis (Fig. 2b) [2, 33, 37, 38]. This phenotype is a consequence of the inclusion of growth factors such as IL2 in culture medium and the use of high nutrient media to maintain effector T cells in culture. Indeed, the ability of IL2 to drive high levels of protein synthesis to sustain T cell size and proliferation is the reason this cytokine is so widely used to generate effector T cells in vitro [39]. In the absence of IL2, in vitro cultured T cells undergo a rapid reduction in protein synthesis, cease dividing and eventually die [4, 37, 39]. The use of other cytokines, such as IL15, to maintain T cells in vitro leads to low rates of protein synthesis and in fact does not promote the production of effector T cells but rather promotes the differentiation of smaller memory-like T cell populations (Fig. 2c) [40, 41].

Effector T cells produce cytokines and cytolytic molecules that drive pathogen clearance and express high levels of effector molecule mRNAs, including those encoding granzyme B and IFNγ. Granzyme B is one of the most highly expressed proteins in effector CD8 T cells [2, 7, 33, 37, 42]. Irrespective of the discrepancy in global protein synthesis rates, granzyme B is highly translated at time points corresponding to both proliferating and nonproliferating effector T cells in vivo [16]. In contrast, the posttranscriptional regulation of cytokine expression has frequently been observed in activated and effector T cells. For example, IFNγ mRNA is highly expressed in effector CD8 T cells during an in vivo response. At early time points, high IFNγ protein expression correlates with high IFNγ mRNA polysome occupancy. However, at the peak of cell expansion, when global protein synthesis rates are low, the amount of IFNγ mRNA associated with the polysome is substantially reduced, corresponding with a reduction in IFNγ protein production and secretion [16]. This finding is consistent with the results of multiple studies showing that IFNγ expression in T cells is exquisitely controlled in a posttranscriptional manner due to its highly regulated 3'UTR [43–45] and potentially through the structure of its 5'UTR [46]. IFNγ is not the only cytokine that is controlled at the posttranscriptional level. IL4 and IL10 expression has been reported to be high at the mRNA level but repressed at the protein level in self-reactive ‘anergic’ CD4 T cells [45], whereas IFNγ, IL4, and IL10 protein levels are increased in activated T cells deficient in the translation repressor PDCD4 [47]. IFNγ and IL17 production can also be repressed by inhibitors of the eIF4F translation initiation complex [17]. Of note, even in translationally active in vitro-generated T cells, pharmacological or TCR stimulation is often required to reveal the cytokine production potential of the T cell population. For the cytokines IFNγ and TNFα, this involves the release of the translational repression of preformed transcripts, followed by de novo mRNA transcription [48]. For IL4 and IL2, de novo mRNA transcription is required for cytokine production [48, 49]. Similar to effector T cells, memory T cells also contain preformed cytokine mRNAs, and recent work demonstrated that the RBP ZFP36L2 repressed IFNγ and TNFα mRNA until cells were retriggered through the TCR [44].

## Regulators of the protein synthesis rate in T cells

T cells undergo major transitions in protein synthesis activity when they are activated, proliferate and differentiate. The immunological drivers of the increased protein synthesis that supports T cell activation, proliferation, and effector function are signals generated by the T cell antigen receptor and inflammatory cytokines [1–3, 5–7, 33, 37, 38]. In this section, we will outline the major factors that control protein synthesis: (1) antigen receptor- and cytokine-driven increases in amino acid availability, (2) the expression and activity of translational repressor proteins, and (3) the posttranslational modification of translation initiation factors.

### Sourcing amino acids—a key regulator of T cell protein synthesis

**Fig. 3:**
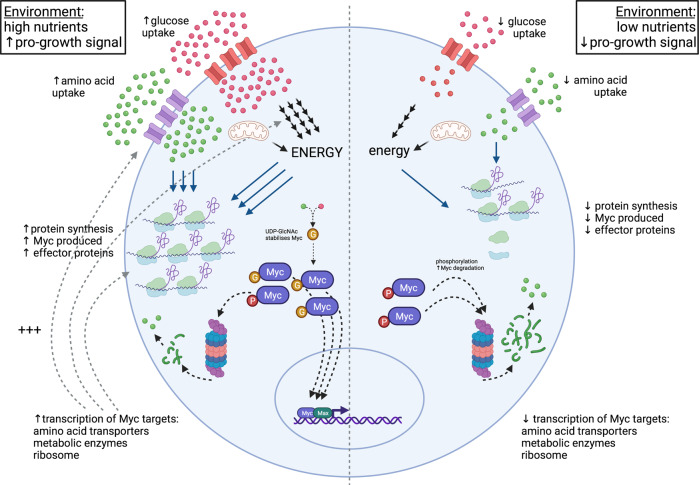
MYC regulation: an example of how feedback among protein synthesis, cell metabolism, and protein degradation controls T cell function. Under high nutrient environments and/or high pro-growth signaling, there are high levels of amino acid and glucose uptake. This fuels high energy (ATP) production, supporting high levels of protein synthesis and the production of UDP-GlcNAc from glutamine and glucose. O-GlcNAcylation at Thr58 stabilizes MYC and prevents its proteasomal degradation. Increased MYC expression promotes the transcription of mRNA for the synthesis of proteins, including amino acid transporters, metabolic enzymes, and ribosomes, thus creating a positive feedforward loop to support a highly biosynthetic environment and sustain high MYC expression. This environment supports the high expression of effector proteins. In contrast, in low nutrient conditions and/or low pro-growth signaling, there is low amino acid and glucose uptake. This results in low energy production and limited fuel and biomolecules for the synthesis of effector proteins. Less MYC is synthesized, and thus, less MYC is O-GlcNAcylated, increasing the proteasomal degradation of MYC. This feedback reduces the MYC-mediated transcription of pro-growth mRNAs

T cells can obtain the amino acids needed to fuel protein synthesis by four main mechanisms: uptake from the environment via amino acid transporters [50, 51], degradation of extracellular proteins internalized by macropinocytosis [52], metabolic pathways that convert other metabolites into amino acids [36, 53], and degradation of intracellular proteins via autophagy [54]. Amino acids can be classified as either essential (His, Iso, Leu, Lys, Met, Phe, Thr, Trp, and Val), meaning they must be acquired from food, or nonessential, encompassing amino acids that can be enzymatically synthesized by cells from other biomolecules, such as glucose and essential amino acids. T cells must import essential amino acids, as they are unable to synthesize them. However, T cells also rely on an extrinsic supply of nonessential amino acids, as they either synthesize insufficient amounts or lack the specific machinery to meet the biosynthetic demand during activation. Thus, T cells are sensitive to the dietary and microenvironment levels of essential and certain nonessential amino acids, and changes in these levels can modulate T cells [36, 55–57]. Here, we focus on the environmental uptake of amino acids, as this appears to be the major mechanism by which T cells source amino acids during activation and proliferation. Naive T cells express very low levels (hundreds or a few thousand copies per cell) of amino acid transporters and take up only low amounts of amino acids [2, 3, 18, 51, 58, 59]. Upon antigen receptor-driven T cell activation, the expression of amino acid transporters is induced within 3–4 h of TCR triggering, supporting the increased amino acid uptake rate [3, 18, 51]. Expression of the key amino acid transporters SLC7A5 (Met, Leu, Iso, Trp, and Val), SLC1A5 (Gln, Ala, Cys, and Met), SLC7A1 (Arg and Lys), SLC38A1 and SLC38A2 (both Gln and Met) increases progressively over the course of T cell activation; these transporters are some of the most induced proteins in the entire proteome, increasing by up to 100-fold by 24 h of antigen activation [2, 3, 18]. Increases in amino acid transport substantially precede any change in the protein content of ribosomes and translation initiation factors [6, 7, 23]. The key immune signals that control amino acid transporter expression are generated by the T cell antigen receptor and cytokines such as IL2. The T cell antigen receptor activates signaling pathways that trigger NFAT and NFκB family protein-driven transcription of the key transcription factor MYC [51, 60, 61]. MYC is required for the expression of *Slc7a5* and *Slc1a5* mRNA, and there is no substantial increase in any amino acid transporter protein upon T cell activation in the absence of MYC [3]. Accordingly, in the absence of MYC, T cells fail to increase amino acid uptake or protein content in response to immune activation [3, 62]. Strikingly, deficiency in a single amino acid transporter, SLC7A5, is sufficient to phenocopy the effects of MYC deficiency, preventing most T cell growth and protein synthesis [3]. In effector T cells, cytokine-induced JAK tyrosine kinase signaling is important for maintaining MYC expression and amino acid uptake [38, 63]. Moreover, JAK inhibition is sufficient to dramatically reduce amino acid uptake and protein synthesis in response to the cytokine IL2 [38, 63]. In both antigen-activated and effector T cells, high levels of amino uptake are required to sustain high levels of MYC protein expression, thus creating a positive feedforward loop involving amino acid uptake, MYC-driven protein synthesis and cell metabolism (Fig. 3) [3, 18, 51, 63].

It has been extensively demonstrated that environmental nutrient availability is key for T cell protein synthesis and growth. Depletion of a number of individual amino acids (Met, Ala, Arg, Asn, and Gln) in T cell culture media has been demonstrated to be sufficient to substantially inhibit T cell growth and protein synthesis during activation and differentiation [18, 51, 53, 64–68]. Similarly, preventing amino acid acquisition by inhibiting the macropinocytosis of serum proteins causes a major defect in cell growth during T cell activation in vitro [52]. Extrinsic amino acid availability is also critical for T cells in vivo. Mouse studies have shown that dietary deficiency of methionine or glycine/serine results in reduced T cell expansion and effector functionality in response to antibacterial or autoimmune responses [36, 55]. Local competition with tumor cells for methionine also suppresses CD8 T cell numbers and effector cytokine production, which can be enhanced by dietary methionine supplementation [56]. Similarly, oral supplementation with arginine has been shown to enhance an antitumor T cell response in mice [58].

Amino acids are needed by T cells as they are the biosynthetic building blocks of protein synthesis. However, other pathways of amino acid metabolism are equally important; for example, methionine is important for producing the methyl donor used for DNA, RNA, and protein methylation [18]; glutamine can be used as an energy source via glutaminolysis [62] or to generate UDP-GlcNAc, the substrate of important glycosyl transferases [69]; and serine is important for purine biosynthesis [36]. T cells can show substantial flexibility in nutrient utilization from metabolic pathways that depend on nutrient availability and enzyme expression, which, in turn, can have a major impact on T cell functionality [70, 71]. For example, inhibition of glutamine metabolizing enzymes using a glutamine analog was recently shown to enhance tumor clearance in a mouse model because energy metabolism within tumor cells was inhibited and because the metabolic adaptation of T cells to enhance glucose and acetate utilization in response to this treatment enhanced their survival and tumor-killing capability [71].

It has, however, been shown that protein synthesis is the major user of amino acids in proliferating cell lines [9]. In particular, the reduced availability of amino acids results in an accumulation of uncharged tRNAs, which can bind and activate the kinase GCN2. GCN2 phosphorylates eIF2a, preventing eIF2 from being recycled to the GTP-bound state required for translation initiation and thus inhibiting global protein synthesis [72, 73]. Whether GCN2 plays a major regulatory role in controlling global protein synthesis in T cells and is a major signaling factor through which amino acid deprivation controls the T cell response are unclear. Several studies have shown that GCN2 contributes to the inhibition of cell cycle progression in T cells and their survival under conditions of nutrient stress, but whether this occurs via its control of global protein translation has not been explored [65, 66, 74]. Furthermore, another study demonstrated that cell cycle inhibition in T cells under amino acid-restricted conditions was not rescued by GCN2 deficiency and that GCN2 deficiency itself affected the cell cycle in a manner independent of its role in amino acid sensing [75]. The integrity of amino-acyl charged tRNA is, however, known to be important for translational regulation in T cells; a recent study demonstrated that the cleavage of amino acid-charged tRNA resulted in reduced protein synthesis in naive and activated T cells [76]. Cellular amino acid levels also regulate the activity of the mammalian/mechanistic target of rapamycin complex 1 (mTORC1) signaling pathway [50, 51, 77, 78]. The role of this pathway in modulating T cell protein synthesis is discussed below.

### Translational repressors—how important are they in T cells?

It is thought that low rates of protein synthesis in quiescent cells can be enforced by proteins known as translational repressors that modulate protein synthesis by inhibiting the function of eIF4F translation initiation complexes. The major translational repressor proteins that have been studied in T cells are eIF4EBP family proteins and PDCD4 (Fig. 1c). These proteins are predominantly thought to be regulated by the serine/threonine kinase mTORC1. The translational repressors eIF4EBP1-3 bind eIF4E in a 1:1 ratio and prevent its association with the scaffold protein eIF4G. Phosphorylation of mouse eIF4EBP1 at residues T36/45 or of eIF4EBP2 at T37/46 by mTORC1 causes dissociation from eIF4E, allowing incorporation into the eIF4F complex [79]. Somewhat unusually, eIF4EBP2 is the more abundant isoform found in T cells [79]. eIF4EBP1 is phosphorylated via mTORC1-sensitive signaling pathways in both TCR- and IL2-stimulated T cells [6, 33, 48, 80]. Ectopic overexpression of constitutively active eIF4EBP1 was shown to strongly inhibit TCR-activated T cell growth and proliferation; however, whether endogenous levels of eIF4EBP1/2 have similar effects in T cells was not determined within this study [79]. Other researchers have reported that eIF4EBP1/2 deletion did not substantially impact mTORC1 inhibition of CD4 T cell proliferation [81]. This raises the question—how important are eIF4EBPs in linking mTORC1 to the regulation of T cell protein synthesis? Measurements of the stoichiometry between eIF4E and eIF4EBP proteins suggested that eIF4EBP proteins are not major regulators of global protein synthesis in T cells. eIF4EBP1-3 proteins must be expressed in excess of eIF4E to repress protein synthesis. However, quantitative mass spectrometry analysis of human naive T cells revealed that eIF4EBP1-3 and eIF4E are at an approximate ratio of only 1:2–5 [6, 7, 42]. Furthermore, eIF4EBP proteins were not detected in mouse naive T cells, which had tens of thousands of copies of eIF4E [2, 3]. In activated and effector T cells, an even more skewed stoichiometry has been measured, with eIF4E outnumbering eIF4EBP proteins by more than 100-fold in mouse effector T cells [2] and by 3–8-fold in human cells [7, 42]. These data indicate that eIF4EBP protein content in T cells is insufficient to prevent eIF4E from associating with the eIF4F complex. Instead, it is likely that eIF4EBP modulation of eIF4F activity has highly selective effects on a subset of eIF4E-sensitive transcripts [13].

Another translation repressor that could be important in T cells is PDCD4, which binds eIF4A at a 1:2 ratio and prevents its association with eIF4G in the eIF4F translation initiation complex [82]. Quantitative mass spectrometry analysis of T cell proteomes revealed that PDCD4 is highly expressed in naive T cells (200,000–400,000 copies per cell) [2, 7]. PDCD4 expression is rapidly reduced in the first few hours of T cell activation [6], resulting in a change in the PDCD4: eIF4A1 ratio from 1:~3 in naive cells to 1:~120 at 24 h in TCR-activated T cells [2]. This rapid reduction in PDCD4 expression in T cells corresponds with increased phosphorylation of Ser76, the RSK1 site [6], and likely also Ser67/71, the S6K1 sites. These phosphorylation events drive the swift proteasomal degradation of PDCD4 [83]. In PDCD4-deficient mice, protein synthesis in total thymocytes or splenocytes showed a clear ~1.5–3-fold increase [47], although whether naive T cells specifically contribute to this translational increase is not clear. In activated T cells (in which PDCD4 content is already relatively low), *Pdcd4* deletion caused a selective increase in the cytokines IFNγ, IL4, and IL10 [47]. In vivo, PDCD4-deficient effector CD8 T cells also exhibited increased IFNγ production in tumor models, resulting in delayed tumor growth [84].

### Control of protein synthesis in T cells: challenging the mTORC1 myth

The importance of matching T cell protein production to the demands of immune effector T cells has prompted a focus on the key signaling molecules that control T cell protein synthesis. In this context, there is a pervasive dogma that the serine/threonine kinase mTORC1, which is activated in T cells in response to antigens, cytokines, and nutrients (in particular, amino acids and glucose), is a major regulator of protein synthesis in T cells. This dogma stems from work on mTORC1 as a regulator of protein synthesis in yeast and nonlymphoid cells [85–87]. However, while it is clear that mTORC1 is a critical regulator of T cell differentiation and controls T cell homing [77, 88], its role in regulating protein translation in T cells is highly selective. Indeed, mTORC1-independent signaling pathways are quantitatively more important for controlling protein synthesis in T cells [1–3, 60], and while immune-activated T cells can increase cell protein mass 3–4-fold, inhibition of mTORC1 only reduced this mass by ~20% [2, 33, 80]. Similarly, the impact of the mTORC1 inhibitor rapamycin on T cell protein translation in an in vivo infection model was modest, with only a small reduction in polysome association seen for several of the ribosome mRNAs measured [16].

How does mTORC1 selectively control T cell protein synthesis? mTORC1 regulates the phosphorylation of numerous eukaryotic translation initiation factors, translational repressors, and ribosomal subunits in activated T cells. For example, mTORC1 controls the phosphorylation of the translational repressors eIF4EBPs and PDCD4 [6]. However, as outlined earlier, none of these individual repressors appear to play a major role in controlling global protein synthesis in T cells. T cell activation is also associated with increased mTORC1-regulated phosphorylation of eIF4B at Ser422 [6], which is reported to increase the interaction of eIF4B with eIF3 and enhance translation initiation [89]. It is also well established that T cell activation induces RSK1- or mTORC1-controlled serine phosphorylation of the small ribosomal subunit protein S6 (RPS6) [90–92]. Indeed, flow cytometry measurements of RPS6 phosphorylation are frequently used to monitor the cellular activity of mTORC1 [51, 80, 90, 91]. Although this phosphorylation may be important in some cells [92], accumulating evidence indicates that it is not a critical switch for protein synthesis in T cells. Salmond et al. demonstrated that T cells in which wild-type RPS6 alleles were replaced with RPS6 alleles with mutations in all five phosphorylatable serine residues activate, grow, proliferate and differentiate normally [90]. Similarly, So et al. demonstrated that T cells deficient for the key RPS6 kinases S6K1 and S6K2 underwent growth and proliferation similar to wild-type cells [79]. A plausible mechanism for a more substantial selective effect of mTORC1 on protein synthesis in T cells involves the pathways that couple mTORC1 to the translational control of 5' terminal oligopyrimidine tract (5'TOP) mRNAs, which include mRNAs encoding ribosomes and translation factors. While the exact regulatory mechanisms are still being determined, it is thought that 5'TOP-containing mRNA is bound by the repressive RBP Larp1, which releases the 5'TOP mRNA upon phosphorylation by mTORC1 [13, 32]. mTORC1-mediated Larp1 phosphorylation has been detected within 2 h of T cell activation [6]; moreover, within 6–24 h of TCR activation, the expression levels of proteins encoded by 5'TOP mRNAs were found to be sensitive to mTOR inhibition [2, 7]. Nevertheless, the salient point is that the impact of mTORC1 inhibition on T cell protein synthesis is limited, selective and context dependent.

These studies highlight that it is important to look beyond mTORC1 signaling when trying to understand how antigen receptors and cytokines control protein synthesis during a T cell immune response. One hypothesis is that T cell protein synthesis is also regulated by the serine/threonine kinases MNK1/MNK2, which phosphorylate eIF4E at Ser209 within minutes of T cell activation [93]; this phosphorylation event selectively controls protein translation in fibroblasts [94]. However, *Mnk1*/*Mnk2* deletion, which ablated eIF4E Ser209 phosphorylation, failed to impact T cell activation, proliferation, or effector cell differentiation, with only specific effects on cytokine production in an in vivo setting [93]. These results thus highlight that many textbook regulators of mRNA translation play only a selective role in regulating protein production in T cells. As discussed previously, a key switch that reproducibly regulates T cell protein synthesis is the antigen and cytokine receptor control of MYC, which drives the expression of amino acid transporters to supply the key building blocks for protein synthesis. Whether there are other critical intracellular signaling molecules that couple the TCR and cytokines, such as IL2, to global control of mRNA translation remains largely unclear.

## How protein degradation changes during a T cell immune response

Howden et al. analyzed how T cell activation shapes T cell proteomes and estimated that ~7–9% of T cell proteins are downregulated as T cells respond to antigen [2]. Proteins whose expression decreases after immune activation include cell cycle inhibitors, transcription factors, and translational repressors. This controlled destruction of proteins that maintain T cell quiescence is essential for T cell activation. Moreover, the balanced activities of protein synthesis and protein degradation are critical for maintaining protein expression levels throughout T cell differentiation. The major pathways by which cellular proteins are degraded include the ubiquitin–proteasome pathway and lysosomal proteolysis during autophagy or endocytosis.

### Proteasomal degradation

Proteins are targeted for proteasomal degradation when they are tagged with a polyubiquitin chain by ubiquitin ligases. Polyubiquitinated proteins are shuttled to the proteasome, where regulatory cap subunits recognize the protein, deubiquitinate it, and unfold it in preparation for proteolytic degradation by the protease activity of the proteasome core subunit proteins [95]. This process is important for T cell protein homeostasis, and the inhibition of proteasome activity results in large-scale cell death [96–98]. Proteasome activity can be regulated by proteasome quantity and composition, posttranslational modifications and the expression of proteasomal activators [95]. The extent to which each of these factors is relevant in T cells during an immune response is not well understood.

T cells predominantly express a specialized form of the proteasome dubbed the “immunoproteasome”, which differs by several subunits from the proteasome in most nonimmune cell types [95, 99]. T cell activation proteomics datasets have revealed a coordinated increase in proteasomal protein expression, scaling with cell growth from ~12 to 24 h post activation [2, 6, 7]. Proteasome protein levels are maintained or increased further in in vitro-generated effector CD8 and CD4 T cell subsets [2, 7]. Examination of proteomics data from proliferating T cells during an in vivo infection showed moderately higher expression of a number of proteasome subunits relative to naive T cells on the same day [36]. A study measuring the proteolytic activity of purified proteasomes of naive versus 48 h in vitro-activated T cells showed an increase in proteolytic enzyme activity upon T cell activation [100], consistent with the increase in proteasome expression. Another study using an activity-based fluorescent probe for detecting proteasomal subunits in intact live cells showed that dividing in vivo CD8 T cells responding to *Listeria*-OVA had high proteasomal activity if they had a memory precursor (CD62LhiCD25lo) phenotype and lower proteasomal activity if they had an effector precursor (CD62LloCD25hi) phenotype at 2.5 days post infection [101]. How this early postactivation activity differs relative to that of naive T cells was not measured; however, the researchers noted that naive T cells and terminal effector CD8 T cells had equivalent proteasomal activity [101]. The molecular impact of changes in proteasome levels and activity in effector T cells is not fully understood, but proteasome control of the expression of TBET, a key transcription factor for T cell effector differentiation, has been proposed [102]. It has also been shown that T cells deficient for the immunoproteasome-specialized subunit LMP7 fail to normally differentiate into effector cells [103].

Is there immune control of proteasomal protein degradation pathways in T cells? One obvious point of control is the phosphorylation of some proteins in immune-activated T cells (e.g., the transcription factor MYC and the translational repressor PDCD4) that targets these proteins for ubiquitination and degradation (Fig. 3) [6, 63, 83]. It may also be pertinent that multiple posttranslational modifications have been reported to modulate proteasome activity, including phosphorylation, polyADP ribosylation, methylation, acetylation, and S-glutathionylation [95]. There is also biochemical evidence for antigen receptor- and IL2-regulated phosphorylation of proteasome subunits [6, 38], but whether these are functionally meaningful in T cells is not known. Indeed, the role of posttranslational modifications of the proteasome subunit in shaping proteasome activity in T cells is largely unexplored, with the exception that there is some understanding of the regulation and function of the E3 ubiquitin ligases that ubiquitinate and target proteins to the proteasome for degradation. This targeted degradation is important for shaping the overall proteome, triggering and dampening cell signaling, and destroying unwanted proteins, which is important for maintaining or rapidly changing T cell fate. The specific targets of E3 ubiquitin ligases and how they control T cell differentiation are beyond the scope of this review, and we refer readers to several good reviews on these topics [104, 105].

### Lysosomal proteolysis—autophagy

The other major mechanism by which proteins are degraded is lysosomal proteolysis, wherein lysosomal fusion with other vesicles allows lysosomal hydrolases to destroy the contents of the vesicle. This process occurs either via endocytosis, whereby the plasma membrane and extracellular proteins are internalized, or via autophagy, whereby cytoplasmic proteins and organelles become encompassed by a double membrane structure called the autophagosome, which fuses with the lysosome [106–108]. For the purposes of this review, we will focus on lysosomal degradation by autophagy, as this process functions to provide amino acids to fuel protein synthesis. Over recent years, it has become evident that autophagy is important for shaping T cell immunity, although the literature is somewhat conflicting regarding when autophagy occurs in T cells and exactly how it is regulated at different stages of the T cell response [106–108]. However, there is a consensus that autophagy is important for generating and maintaining memory CD4 and CD8 T cells. Multiple studies using genetic deletions of important autophagy proteins (ATG5 and ATG7) have revealed defects in memory cell numbers and recall responses [109–112]. Furthermore, autophagy is critical for naive T cell survival; studies using conditional genetic deletion of autophagy proteins (VPS34, ATG3, ATG5, and ATG7) showed reduced peripheral naive T cell numbers, which were attributed to a failure to remove damaged mitochondria [113–115] [109, 116]. In human naive and memory CD4 T cells, proteomic studies have found that the autophagy cargo receptor SQSTM1 is one of the most rapidly turned over proteins [7]. When naive T cell protein synthesis was inhibited, proteasomal inhibition did not rescue the loss of SQSTM1 expression, supporting the idea that autophagy occurs in these cells. Not all of the proteins that were turned over in a proteasome-independent manner were mitochondrial [7], providing clues as to other targets of autophagy in naive T cells.

Examinations of T cell proteomes have shown a net increase in components of the autophagy pathway after 24 h of T cell activation [2, 7]. Several studies have monitored the expression of lipidated LC3B, a key protein involved in autophagy substrate selection, and reported increases in autophagic vesicle expression upon TCR activation and effector differentiation [117–119]. This increase in autophagy-mediating proteins in activated T cells could correspond with an enhanced capability for autophagy but could also indicate that autophagic flux is reduced and proteins are no longer being destroyed. More accurate monitoring of autophagic flux in T cells over the course of an in vivo LCMV infection suggested that autophagy was reduced in activated T cells [112]. The precise timing of autophagy regulation in T cells is thus somewhat controversial, reflecting the difficulty in interpreting autophagy assays [120]. Nevertheless, it is evident that dynamic changes in autophagy do occur during T cell activation and that autophagy is important for T cell participation in adaptive immune responses.

## T cell energy metabolism—what have we learned from proteomics studies?

Following antigen receptor engagement, very fast changes occur in multiple cellular metabolism pathways. Activated T cells increase glycolysis and mitochondrial OXPHOS to produce ATP while decreasing mitochondrial fatty acid oxidation (FAO) [36, 43, 62]. How T cells control glucose and fatty acid metabolism over the course of an immune response has been extensively reviewed [121–123]. Here, we will briefly summarize how T cell glucose metabolism changes during immune activation (illustrated in Fig. 2), incorporating new insights from high-resolution mass spectrometry before discussing crosstalk between glucose metabolism and protein synthesis and degradation.

The increase in the T cell glycolytic rate is relatively rapid, detectable within a few minutes of TCR stimulation, and this rate continues to increase over hours of immune activation [62, 124]. Naive T cells already contain a large amount of glycolytic enzymes [2, 3, 7], but the expression of most glycolytic enzymes increases after ~8–12 h of immune activation [5–7]. One rate-limiting step in glycolysis is glucose supply [33, 125, 126]. The glucose transport capacity of naive T cells is very low [69, 80, 127], but increases in glucose transport capability have been detected within 2 h of T cell activation (Linda V. Sinclair, personal communication), and high levels of glucose transport have been detected in effector cells [33, 69, 80, 127]. The sustained increase in the glucose transport capacity of immune-activated T cells is mediated by increased glucose transporter expression. T cells express two glucose transporters, GLUT1 (SLC2A1) and GLUT3 (SLC2A3). The expression of these proteins is very low in naive T cells but high in antigen-activated and effector T cells [1–3, 7, 33]. Studies have typically not measured a net increase in total glucose transporter expression levels until 8–12 h post TCR stimulation [6, 7]; however, it is proposed that glucose transporters shuttle from intracellular vesicles to the cell surface during T cell activation [125]. In this context, it was recently noted that the protein TXNIP, which drives glucose transporter endocytosis, is rapidly destroyed upon T cell activation [6, 7]; this process could regulate early glucose uptake. However, glucose import and lactate export must both increase for increased glycolytic flux. It is thus striking that immune-activated T cells rapidly increase the expression of the lactate exporters MCT1 (SLC16A1) and MCT4 (SLC16A3) [6, 7].

Increased mitochondrial OXPHOS has also been measured within hours of T cell activation [5] and has been shown to increase as T cells activate and differentiate [36, 43]. These changes in T cell metabolism are supported by dramatic mitochondrial remodeling and mitochondrial biogenesis [5, 6]. Mitochondria number and protein content do not increase within the first few hours of T cell activation but gradually increase from ~9 h onwards [5]. In addition to glucose, OXPHOS can be fueled by amino acids such as glutamine during T cell activation [62]. Glutamine transporter expression increases during early T cell activation [3] and may contribute to the early increase in OXPHOS observed in T cells. Mitochondrial protein content does not scale with only mitochondrial number or total cell size upon T cell activation; mitochondrial proteins make up a higher percentage of the total cellular protein content at 24 h in in vitro immune-activated and effector T cells than in naive T cells [2]. Furthermore, select mitochondrial proteins, such as those involved in one-carbon metabolism, which generates metabolites for de novo purine biosynthesis and controls glutathione levels, are rapidly and disproportionately increased compared to other mitochondrial components [5].

## Crosstalk between glucose metabolism and protein synthesis and degradation

Protein synthesis is a metabolically demanding process. It has been estimated that ~5 ATP per peptide bond or ~2300 ATP per typical protein synthesized are required [10]. This requirement equates to the energy from ~1100 molecules of glucose processed through glycolysis or ~60–70 molecules of glucose for OXPHOS if these processes were 100% efficient. The intrinsic link between ATP production and protein synthesis was demonstrated in an elegant technology paper by Arguello et al., who exploited the ATP dependence of protein synthesis to measure the metabolic profile of single cells [128]. In this study, protein synthesis in T cells was assayed using a single flow cytometry assay that quantifies the incorporation of a puromycin analog into nascent protein chains in the ribosome. This enabled the use of inhibitors of either glycolysis or oxidative phosphorylation to assess the contribution of ATP produced by either pathway to protein production. This work showed that the inhibition of energy metabolism across all T cell differentiation states caused a strong decrease in protein synthesis, and the authors concluded that protein synthesis in TCR-activated and effector T cells was more dependent on glycolysis, whereas oxidative phosphorylation was more important for driving protein synthesis in naive and memory T cells.

Autophagy can be triggered by nutrient deprivation in multiple cell types. Protein degradation via autophagy generates more resources and energy than are utilized in the process [54]. Very little is known about crosstalk between energy metabolism and autophagy in T cells, although several studies have noted that a decrease in autophagy is linked to an increase in glycolysis [129–131] and have implicated glycolytic enzymes as potential targets of autophagy [130]. Whether these data imply a mechanism by which environmental nutrient stress feeds back to dampen metabolic pathways that support high levels of biosynthesis in T cells requires further study. Protein degradation via the proteasome is a net consumer of ATP, although the amount as a proportion of the total cellular energy usage is thought to be relatively small [10]. In cultured neurons, inhibition of complex I of the electron transport chain reduced proteasome activity [132], but the extent to which a reduction in ATP inhibits proteasomal activity in T cells is unclear. There is, however, evidence that protein degradation via the proteasome can feedback to control cellular metabolism in T cells. For example, treatment of activated CD8 T cells with a proteasome inhibitor increased glycolysis, whereas T cells treated with a proteasome activator showed decreased glycolysis [101]. One explanation for this phenomenon is that the expression of glucose transporters, lactate transporters, and glycolytic enzymes in T cells is controlled by the transcription factor HIF1a [80]. HIF1a has a very short half-life in effector T cells because it is ubiquitinated by the VHL-containing ubiquitin ligase complex and then targeted for proteasomal degradation. Proteosome inhibition would thus increase the expression of HIF1a, which is known to drive glycolysis in effector T cells [80]. Changes in proteasome activity could also regulate the expression of MYC, another transcription factor that controls T cell metabolism [3, 62]. In T cells, MYC has a short half-life because it can be rapidly degraded by the proteasome. A reduction in proteasomal activity thus substantially increases MYC expression [63]. Moreover, as illustrated in Fig. 3, the expression of MYC is an interesting example of how the nutrient environment can shape the expression of a protein by balancing protein synthesis and degradation. MYC degradation is controlled by phosphorylation at Thr58, which helps recruit ubiquitin ligases, hence targeting MYC for proteasomal degradation [63]. However, MYC can be O-GlcNAcylated at Thr58, which prevents Thr58 phosphorylation and MYC degradation, allowing this transcription factor to accumulate. The rate of MYC O-GlcNAcylation is controlled by the supply of glutamine and glucose, which are metabolized via the hexosamine biosynthetic pathway to produce UDP-GlcNAc, the donor substrate for O-GlcNAc transferase, which catalyzes the addition of O-linked-β-N-acetylglucosamine (O-GlcNAc) to MYC Th58 [69]. Thus, a drop in either glucose or glutamine availability results in reduced MYC GlcNAcylation, the subsequent loss of MYC expression and, consequently, the loss of expression of key MYC-controlled metabolic proteins. In this context, and as previously discussed, MYC-controlled expression of amino acid transporters is an essential switch for controlling protein production.

## Conclusion

The precise regulation of protein synthesis and degradation shapes the functional capabilities of T cell immune responses. Technological advances have revealed the constant dynamic turnover of proteins in both naive and effector T cells and the balance between protein synthesis and degradation that is key for controlling protein expression. There have also been major advances in identifying some crucial checkpoints for T cell protein synthesis, including the importance of the regulation of amino acid transporter expression and immune control of ATP production. There is, however, more to learn, particularly about how diet and competition with other cells shape nutrient availability to T cells and how signaling molecules and environmental nutrients selectively impact T cell protein expression. Understanding how T cells prioritize protein production and which proteins they will degrade under conditions of energy and nutrient stress will be of particular value for efforts to refine anticancer T cell therapies and develop T cell tissue-targeted therapies for inflammatory disease progression. However, there are now sensitive and quantitative technologies for measuring protein synthesis and T cell proteomes that will facilitate our understanding of these key issues.
